# Complete Solubilization and Purification of Recombinant Human Growth Hormone Produced in *Escherichia coli*

**DOI:** 10.1371/journal.pone.0056168

**Published:** 2013-02-07

**Authors:** Min-Ji Kim, Hyun Soo Park, Kyung Hye Seo, Hyo-Jin Yang, Sook-Kyung Kim, Jun-Hyuk Choi

**Affiliations:** 1 Center for Bioanalysis, Department of Metrology for Quality of Life, Korea Research Institute of Standards and Science, 1 Doryong-dong, Youseong-gu, Daejeon, South Korea; 2 Department of Bio-Analytical Science, University of Science & Technology, Daejion, South Korea; Queensland Institute of Medical Research, Ustralia

## Abstract

High-level expression of recombinant human growth hormone (hGH) in *Escherichia coli (E. coli)* leads to the formation of insoluble aggregates as inclusion bodies devoid of biological activity. Until recently, significant efforts have been made to improve the recovery of active hGH from inclusion bodies. Here, we developed an efficient procedure for the production of completely soluble hGH by minimizing the formation of inclusion bodies and optimizing protein purification conditions. Under the newly established conditions we were able to obtain most of the total hGH in the soluble fraction. We show that the soluble protein can be efficiently purified in high yield by a series of chromatographic procedures. We analyzed the resulting hGH using various analytical techniques such as reversed-phase high-performance liquid chromatography (RP-HPLC), size-exclusion chromatography (SEC), matrix-assisted laser desorption ionization time-of-flight (MALDI-TOF) mass spectrometry, and circular dichroism (CD). These multiple analyses support the conclusion that we obtained highly pure hGH with the expected molecular mass and intact secondary structure. The biological activity of purified hGH was also confirmed by evaluating its growth-promoting effect using a cell proliferation assay. Taken together, we describe a straightforward strategy for the production of completely soluble and biologically active hGH in *E. coli.*

## Introduction

Human growth hormone (hGH) is a single-chain polypeptide containing 191 amino acid residues and is synthesized in the pituitary gland. It is one of the most important hormones in the human body due to its pivotal role in a variety of biological functions, including cell proliferation and metabolism [1]. Endogenous hGH is a non-glycosylated protein [2], and consequently recombinant forms of hGH have been extensively produced in prokaryotic expression systems [3], [4]. However, high-level expression of hGH in *E. coli* results in insoluble protein aggregates as inclusion bodies that are often observed with many other eukaryotic proteins [3], [4], [5]. Therefore, the aggregated hGH requires solubilization and refolding steps prior to purification by chromatography, and the overall protein recovery is significantly affected by the efficiency of these pre-purification steps. In general, the aggregated proteins need to be solubilized using high concentrations of denaturants such as urea or guanidine hydrochloride (GnHCl), followed by removal of denaturants for protein refolding. In many cases, however, the overall yield of biologically active protein from inclusion bodies is very low, and these purification procedures are often costly and time–consuming [6]. Therefore, considerable efforts have been made to increase the efficiency of solubilization and refolding as a means of improving the overall recovery of biologically active hGH from inclusion bodies [7], [8], [9], [10]. An alternative approach that has been developed in order to prevent the formation of inclusion bodies relies on the secretion of hGH into the bacterial periplasmic space using a signal peptide [11], [12], [13]. The yields associated with periplasmic expression using different expression constructs have been extensively investigated under various conditions [14].

In the present study, we describe a much simpler approach to maximize the solubility of hGH expressed in bacterial cytoplasm by optimizing the conditions for protein expression, extraction and purification.

## Materials and Methods

### Gene cloning of hGH

The hGH gene (GenBank Accession No. K02382.1) construct was amplified from human cDNA by the polymerase chain reaction (PCR) using forward primer 5`-gcggctagcatgttcccaaccattcccttatcc-3` and reverse primer 5`-gcgctcgagctagaagccacagctgccctc-3` (NheI and XhoI restriction enzyme sites underlined, respectively). The hGH gene was cloned into pET-28b (Novagen, Madison, WI, USA) expression plasmid to produce recombinant hGH with a hexahistidine tag and a thrombin cleavage site at the N-terminus (His-hGH). For untagged hGH, the gene construct was amplified by PCR with forward primer 5`-gcgccatggcgatgttcccaaccattcccttat-3` and reverse primer 5`-gcgctcgagctagaagccacagctgccctc-3` (NcoI and XhoI restriction enzyme sites underlined, respectively). The PCR product was digested with NcoI and XhoI, and ligated into NcoI and XhoI restriction sites of pET-28a (Novagen) expression vector to produce recombinant hGH without a hexahistidine tag at the N-terminus (untagged hGH). The nucleotide sequences of inserts were verified by automatic sequencing.

### Solubility assay

Plasmids pET28-His-hGH and pET28-hGH were transformed into *E. coli* BL21 (DE3) cells for protein expression. A 10 ml aliquot of an overnight culture was seeded into 500 ml of fresh LB (Luria-Bertani) medium (10 g Bacto tryptone, 5 g yeast extract and 10 g NaCl per liter of solution) containing 50 µg/ml kanamycin and grown to an OD_600_ of about 0.6 at 37°C. Recombinant protein expression was induced with 1 mM isopropyl-β-D-thiogalactopyranoside (IPTG), and cells were further grown at different temperatures where indicated. The cells were then harvested by centrifugation at 6,000 g for 20 min, and cell pellets were suspended in lysis buffer (50 mM Tris-HCl, pH 8.0, 0.5 mM EDTA, 1 mg/ml lysozyme) containing 1× protease inhibitor cocktail (Roche, Barcelona, Spain). During protein extraction, solubility assays were performed using different buffer additives and buffer volumes (up to 2 ml for 30 mg cell pellets). Following incubation on ice for 30 min, cells were sonicated at 200 W (10 times for 10 s) using an Uibra cell TM sonicator (Sonics & Materials, Newtown, CT, USA) and then centrifuged at 10,000 g for 20 min. The soluble (supernatant) and insoluble (pellet) fractions were analyzed by SDS-PAGE and Coomassie blue staining. The level of protein was quantified using ImageQuant™ TL 5.2 analysis software (GE Healthcare, Piscataway, NJ, USA) after scanning gels on a V799 scanner (EPSON, Long Beach, CA, USA). Averages from at least three independent experiments were graphed and presented as mean ± standard deviation, except for induction at 37°C.

### Purification of recombinant hGH

Through extensive solubility assays, the protein expression and extraction conditions were optimized as follows. *E. coli* BL21 (DE3) cells expressing recombinant hGH (untagged hGH and His-hGH) were grown in a 1 L baffled flask containing 250 ml of culture medium and induced at 16°C for 16 h. Harvested cells were suspended in 25 ml of lysis buffer (50 mM Tris-HCl, pH 8.0, 0.5 mM EDTA, 0.1% Triton X-100, 1 mg/ml lysozyme, 1× protease inhibitor cocktail), disrupted by sonication, and then centrifuged at 10,000 g for 20 min. For purification of His-hGH, the supernatant was applied onto a 5 ml Ni-NTA agarose column (Qiagen, Valencia, CA, USA). The column was washed with 3 column volumes of washing buffer (1× PBS, pH 7.4, containing 150 mM NaCl, 10 mM imidazole, 0.1% Triton X-100, 10% glycerol) and eluted with elution buffer (1× PBS, pH 7.4, containing 150 mM NaCl, 200 mM imidazole, 0.1% Triton X-100, 10% glycerol). Fractions containing His-hGH were pooled and dialyzed against buffer containing 50 mM Tris-HCl (pH 8.0) and 10% glycerol. Dialyzed fractions were further purified by anion-exchange chromatography on a 1 ml Mono Q 5/50 GL column (GE Healthcare), and eluted with a 10-column volume linear gradient of 0–0.5 M NaCl. Fractions forming the major peak containing His-hGH were pooled and finally purified by gel-filtration using a HiLoad 26/30 Superdex 200 column (GE Healthcare) equilibrated with buffer containing 50 mM Tris-HCl (pH 8.0), 150 mM NaCl and 10% glycerol. For purification of untagged hGH, the supernatant was dialyzed against binding buffer containing 50 mM Tris-HCl (pH 8.0) and 10% glycerol. Dialyzed lysates were loaded onto a 20 ml Hiprep DEAE FF 16/10 column (GE Healthcare) and eluted with a 10-column volume of 0 – 1 M NaCl gradient. Fractions containing hGH were pooled and dialyzed again. Dialyzed fractions were purified by anion-exchange and gel-filtration chromatography under the same conditions as described above. Purified proteins were dialyzed against storage buffer (10 mM Na_2_HPO_4_, pH 7.4, 0.5% glycine, 2.25% mannitol). Note that all purification procedures were performed at 4°C. Protein concentration was determined by the Bradford and bicinchoninic acid (BCA) assays using BSA as a standard [15]. Protein purity was initially monitored by SDS-PAGE and silver staining, and later by more advanced methods as specified below.

### Characterization of purified proteins

Protein purity was examined by RP-HPLC using a Kinetex C18 column (2.6 µm, 150×2.10 mm; Phenomenex, Torrance, CA, USA) [16]. Analytes were eluted using a linear gradient from 28% in buffer A (0.1% trifluoroacetic acid in H_2_O) to 100% in buffer B (0.1% trifluoroacetic acid in acetonitrile) at 40°C. The flow rate was 0.2 ml/min and detection was achieved by monitoring the UV absorbance at 220 nm.

Analytical size exclusion chromatography was performed on an AKTA FPLC system using a Superdex 75 10/300 GL column (GE Healthcare). The purified hGH was injected into the column using a 100 µl sample loop with an injection volume of 100 µl. Protein was eluted isocratically with 50 mM Tris-HCl (pH 8.0), 150 mM NaCl and 10% glycerol at a flow rate of 0.8 ml/min. The reliability of the analysis was confirmed by at least two independent measurements under the same conditions. Detection was achieved by monitoring the UV absorbance at 280 nm.

MALDI-TOF mass spectrometry was performed on an Autoflex III Smartbeam device (Bruker Daltonics, Billerica, MA, USA) [16]. Sample was mixed with the same volume of MALDI matrix (10 mg/mL of α-cyano-4-hydroxycinnamic acid) and spotted on a MALDI target plate. External calibration was performed with a Peptide and Protein MALDI-MS Calibration Kit (Sigma, St. Louis, MO, USA). Mass spectra in the m/z range of 15000–45000 were acquired in the positive ion mode.

Circular dichroism (CD) measurements were performed at 25°C on a J-815 circular dichroism spectropolarimeter (Jasco, Tokyo, Japan) using a quartz cuvette with path length 0.1 mm to measure the far-UV range [17]. Spectra were recorded over a wavelength range of 200–250 nm with bandwidth 0.1 nm, scanning speed 50 nm/min, and 10-s response time. Control hGH was purchased from LG Life Science (Daejeon, South Korea) and analyzed as a control under identical conditions [18].

### Nb2-11 cell culture

The PRL-dependent rat T-lymphoma cell line, Nb2-11 cells (Sigma) were cultured in RPMI 1640 medium (Gibco/Invitrogen, Grand Island, NY, USA) supplemented with 10% fetal bovine serum (FBS) (Gibco/Invitrogen), 10% horse serum (HS) (Gibco/Invitrogen) and 1% penicillin-streptomycin (Gibco/Invitrogen) at 37°C in a humidified atmosphere containing 5% CO_2_ [19]. Cell proliferation was determined using MTS assays [20]. Briefly, cells were harvested, rinsed in culture medium without FBS, and then incubated for 48 h in 96-well plates at 20,000 cells/ml (100 µl/well) in the presence of various amounts of purified hGH. Following incubation, 20 µl of the MTS reagent (Promega, WI, USA) was added to each well, and cells were incubated for 2 h. The absorbance was recorded on a microplate reader (Bio-Rad, CA, USA) at a wavelength of 490 nm. Cell numbers were determined using a standard curve plotted from a linear relationship between cell number and absorbance.

## Results

### Solubilization of recombinant hGH under various conditions

**Figure 1:**
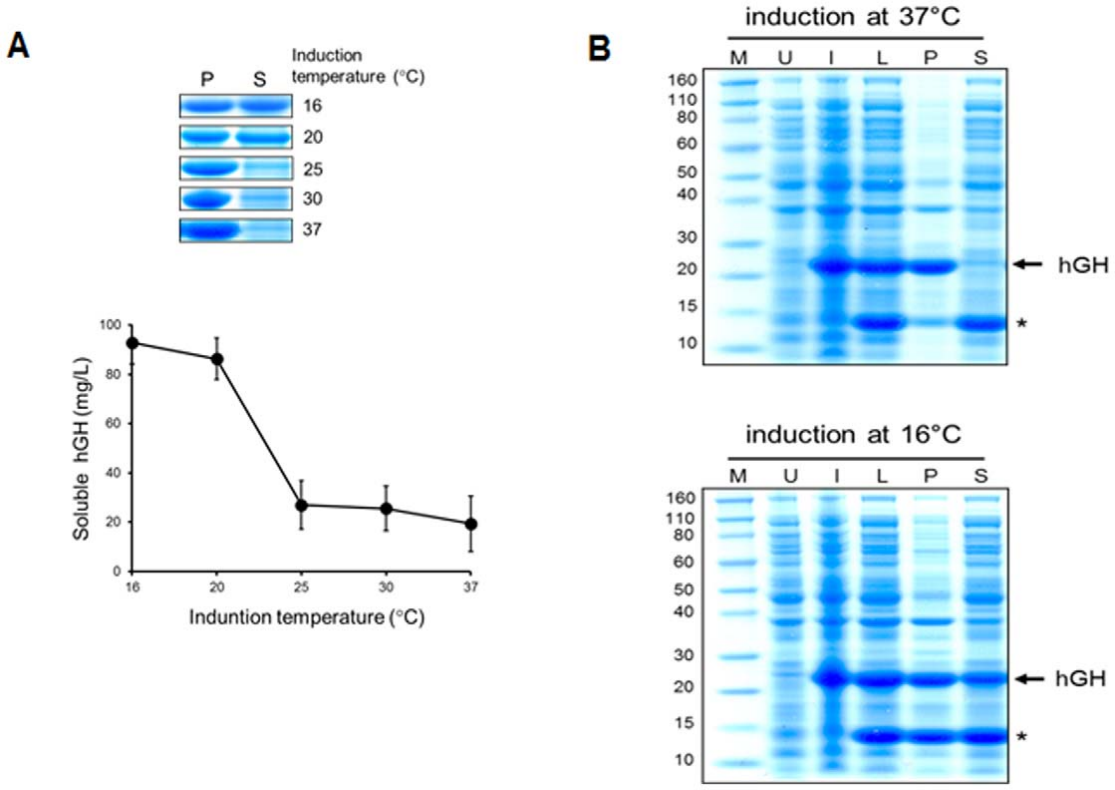
Solubility comparison of recombinant hGH expressed at different temperatures. (A) The levels of insoluble versus soluble hGH upon induction at various temperatures. *E. coli* BL21 cells expressing recombinant hGH were grown to an OD_600_ of 0.6 at 37°C and then induced at the indicated temperatures. Cells were lysed, and the pelleted (P) and soluble (S) fractions were analyzed by SDS-PAGE and Coomassie blue staining (upper panel). The levels of soluble hGH were quantified and graphed by a densitometry assay using ImageQuant™ TL 5.2 analysis software (*bottom*). The values are plotted as mean ± standard error. (B) Increased solubility of hGH expressed at reduced temperature. Protein samples obtained with induction at either 37°C (*upper*) or 16°C (*bottom*) were separated on a 4–12% SDS-PAGE and visualized by Coomassie blue staining. U, uninduced; I, induced; L, lysis; P, pelleted; S, soluble; asterisk; lysozyme.

In agreement with previous studies, we confirmed significant accumulation of insoluble protein aggregates as inclusion bodies upon high-level expression of hGH in *E. coli* (Figure 1). We observed that most of the hGH was found in the insoluble fraction (lane P; pellet fraction), and there was a very small amount of soluble hGH (lane S; soluble fraction). To increase the yield of soluble protein, the protein expression conditions can be optimized by varying induction temperatures, inducer concentrations and media compositions [21]. In particular, protein expression at low temperature often significantly improves the solubility of recombinant proteins [22]. Indeed, we observed that induction at lower temperatures (16–20°C) significantly increased the amount of soluble hGH without modifying our protein extraction method (Figure 1A), indicating that a slow induction of hGH efficiently reduced the formation of inclusion bodies. We observed efficient expression of hGH with a 5-fold increase in solubility upon induction at 16°C for 16 h (Figure 1B), and thus those conditions were used in subsequent experiments. We also determined whether the formation of inclusion bodies could be reduced by lowering IPTG concentration (to 0.1 mM). The results indicate that reducing IPTG concentration is not as effective as lowering induction temperature at preventing the formation of inclusion bodies under our conditions (Figure S1).

**Figure 2:**
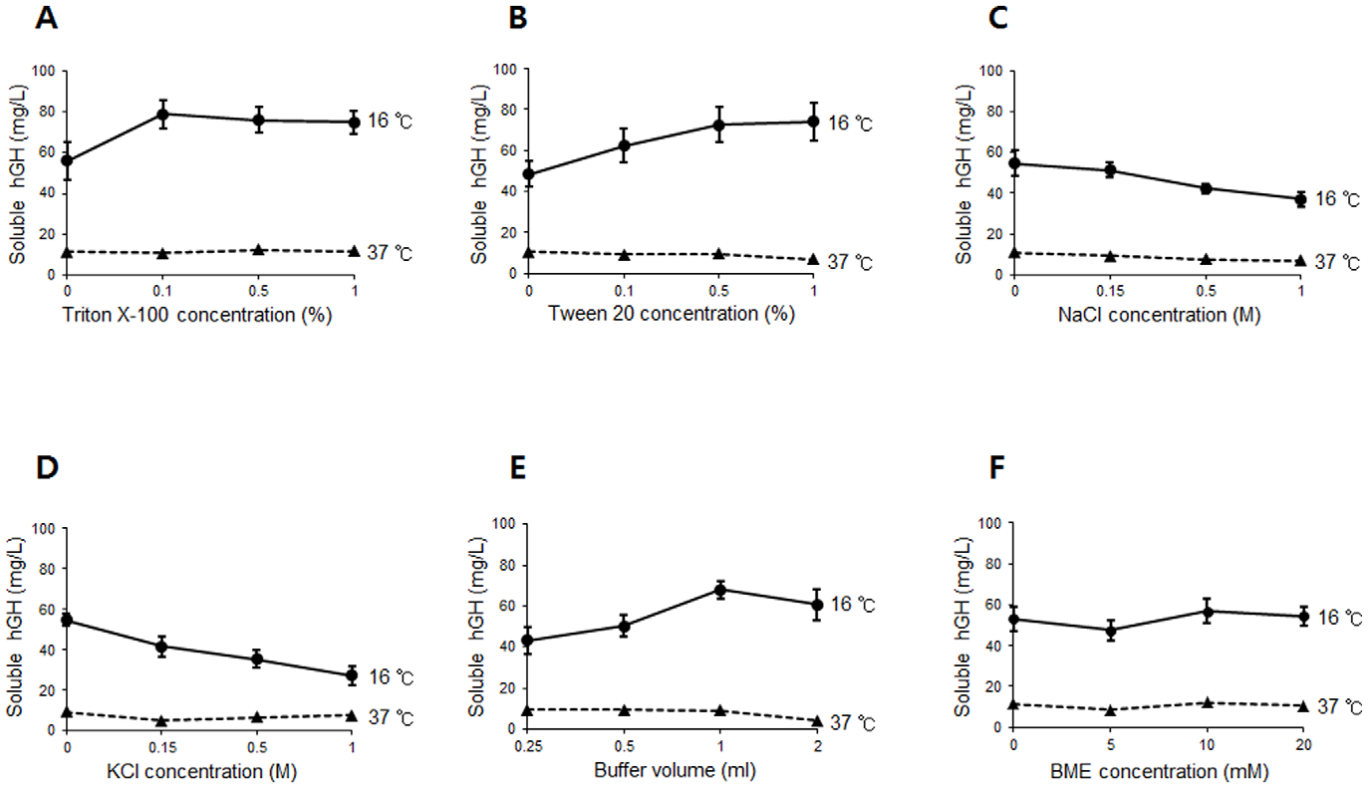
Solubilization of hGH under various extraction conditions. The solubility of hGH, expressed either at 37°C or 16°C, was determined by evaluating the effects of Triton X-100 (A), Tween 20 (B), NaCl (C), KCl (D), buffer volumes (E), and β-mercaptoethanol (F). The levels of soluble hGH were quantified and graphed as described in Figure 1.

As a substantial amount of hGH was still found in the insoluble fraction, we examined whether the pelleted hGH could be recovered by optimizing protein extraction. Although the formation of inclusion bodies may be further reduced by optimizing environmental factors such as culture media [21] or genetic engineering [23], [24], we reasoned that the observed protein aggregation may involve some other factors such as non-specific protein interactions. To reduce such interactions and facilitate protein solubility, a number of useful buffer additives have been identified and efficiently implemented [25]. We first tested the effects of mild non-ionic detergents including Triton X-100 and Tween 20 since they are unlikely to disrupt protein structure. As shown in Figure 2A, the use of Triton X-100 significantly increased the solubility of hGH expressed at low temperature. This non-ionic detergent was very efficient at concentrations as low as 0.1%. The addition of Tween 20 also increased the solubility, but appeared less efficient than Triton X-100 (Figure 2B). Next, we determined the effect of salts on hGH solubility as the solubility of protein is often dependent on the salt concentration. Interestingly, the results show that the addition of NaCl stimulated protein aggregation to some extent (Figure 2C). The observed effect was not specific to NaCl, as even more dramatic effect was observed with KCl (Figure 2D). Although 150 mM salts appeared to have marginal effects (especially NaCl, the more commonly used salt), the solubility of hGH seems to be sensitive to such additives at higher concentrations. Since protein concentration is another important factor in protein aggregation, we assessed protein solubility using different buffer volumes. As expected, the risk of protein aggregation was significantly decreased by appropriate dilution (Figure 2E). Although reducing agents often prevent protein aggregation by inhibiting the formation of non-native intermolecular disulfide bolds, we observed no significant effect of β-mercaptoethanol (Figure 2F).

It should be noted that we observed essentially no effect of the buffer additives on the solubility of hGH expressed at higher temperature (37°C), indicating that irreversible formation of inclusion bodies resulted in insoluble pellets (Figure 2A–F).

### Purification of soluble hGH

**Figure 3:**
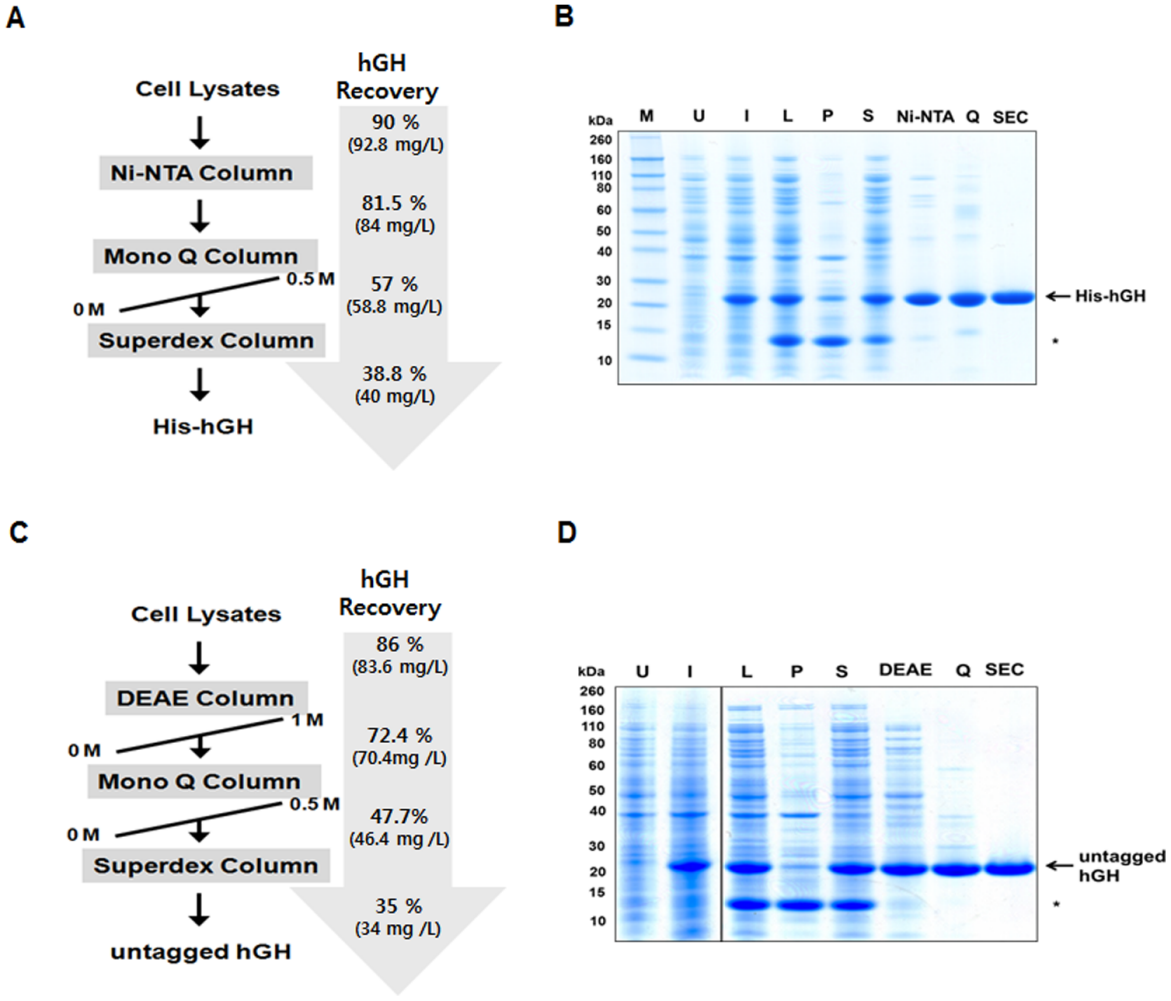
Purification of recombinant hGH under optimized conditions. (A) Scheme of His-hGH purification from *E. coli*. The arrow indicates the overall yield of hGH at each step in the purification process. (B) Purification of His-hGH. Elutions from each column were separated on a 4–12% SDS-polyacrylamide gel and analyzed by Coomassie blue staining. (C) Scheme of His-hGH purification from *E. coli*. (D) Purification of untagged hGH. Elutions were analyzed as in (B).

Based on the results described above, we optimized the conditions for protein expression and the buffer solution for protein extraction. The protein was induced at 16°C for 16 h and extracted using the optimized buffer solution (50 mM Tris-HCl, pH 8.0, 0.5 mM EDTA, 0.1 % Triton X-100, 1 mg/ml lysozyme, 1×protease inhibitor cocktail) and buffer volume (1 ml for 30 mg of pellets). Under these conditions, we observed greatly improved solubility of recombinant hGH (Figure 3). Significantly, the yield of soluble hGH was estimated to be up to 95% of the total hGH expressed in *E. coli*.

As we obtained highly soluble hGH, the protein was subsequently purified by Ni-NTA affinity, anionic exchange, and size-exclusion chromatography (Figure 3A). SDS-PAGE analysis shows a clear, single band with a molecular mass of about 25 kDa when eluted from the final column (Figure 3B). The results for the purification indicate that we achieved efficient purification of hGH with high yield (>40%) and high purity (>97%) under our conditions (Table S1).

To ascertain that the established procedure is not specific to the tagged version of hGH that harbors some additional amino acid residues including the hexahistidine sequence and a protease cleavage site, we produced an untagged form of hGH and confirmed the high level of protein solubility under identical conditions (Figure 3C). While optimizing purification steps for untagged hGH, we found that the consecutive application of a weak followed by a strong anion exchange resins (DEAE and MonoQ respectively) and size-exclusion chromatography produced very high yields of pure hGH (Figure 3D). The results for the purification are summarized in Table S2.

Taken together, these results indicate that we established an efficient purification procedure for soluble hGH in *E. coli* using optimized conditions in which protein aggregation was minimized.

### Characterization of purified hGH

**Figure 4:**
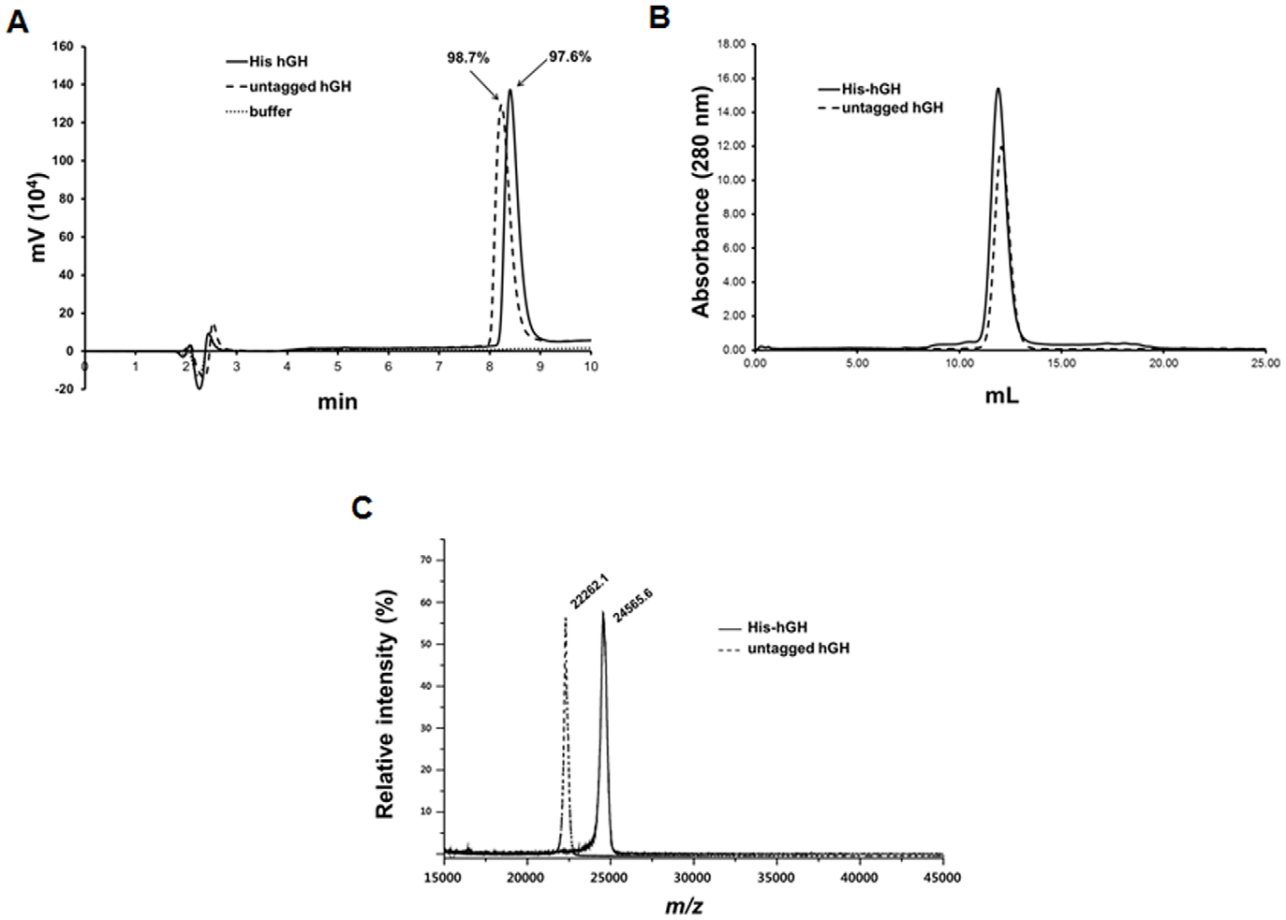
Characterization of purified hGH. (A) RP-HPLC chromatograms of purified hGH. Separation was performed by isocratic elution using trifluoroacetic acid-water-acetonitrile. Vertical axis units represent mV detector output. (B) Analytical size exclusion chromatography (SEC) of purified hGH. (C) MALDI-TOF mass spectrometry analysis of purified intact hGH. Spectra were acquired over the *m/z* range 15000–45000 Da in the positive ion mode.

**Figure 5:**
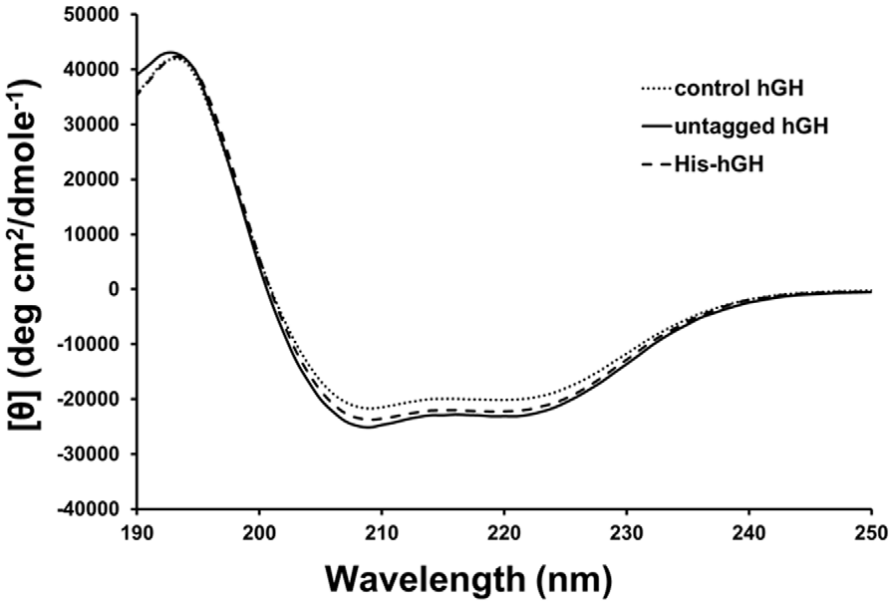
Circular dichroism (CD) analysis of purified hGH. The CD spectra of control hGH, His-hGH and untagged hGH were scanned in the UV range 190–250 nm.

Upon purification, the purified proteins were analyzed by several analytical methods, including RP-HPLC, size-exclusion chromatography, MALDI-TOF mass spectrometry, and circular dichroism (CD). RP-HPLC analyses of the two purified proteins, His-hGH and untagged hGH, showed that they were eluted as a single peak near 8 min and there was no detectable contaminant (Figure 4A). The purity of His-hGH and untagged hGH was estimated to be 97.6% and 98.7%, respectively. The minor peaks near 2.5 min are thought to have been caused by the buffer solution. Next, we performed analytical size-exclusion chromatography (SEC) to determine the molecular mass of the purified proteins (Figure 4B). The results show that their molecular weights were approximately 21.3 kDa (His-hGH, expected: 24.612 kDa) and 20.3 kDa (untagged hGH, expected: 22.246 kDa). Once again, we observed only one major peak for both proteins with no other significant protein peaks, indicating their high purity. As the molecular weights determined by SEC appeared slightly lower than the expected values, we wanted to determine their molecular masses more accurately. To this end, we analyzed the proteins using MALDI-TOF mass spectrometry. As shown in Figure 4C, the measured molecular masses of His-hGH and hGH were 24,565 Da and 22,262 Da, respectively, and these values are very close to the expected masses. Finally, to determine the structural integrity of the purified proteins, structural characterization was performed by circular dichroism (CD) analysis. As shown in Figure 5, we found that the two CD spectra of the purified proteins were very similar to that of the control hGH. In agreement with previous reports [11], [18], we clearly detected a positive peak at 195 nm and a negative peak with a minimum at 208 nm and a shoulder at 222 nm, which are typical characteristics of a structure with high α-helical content. The results suggest that our optimized procedure for protein expression, extraction and purification did not disrupt the structural integrity of the proteins.

### Biological activity of purified hGH

**Figure 6:**
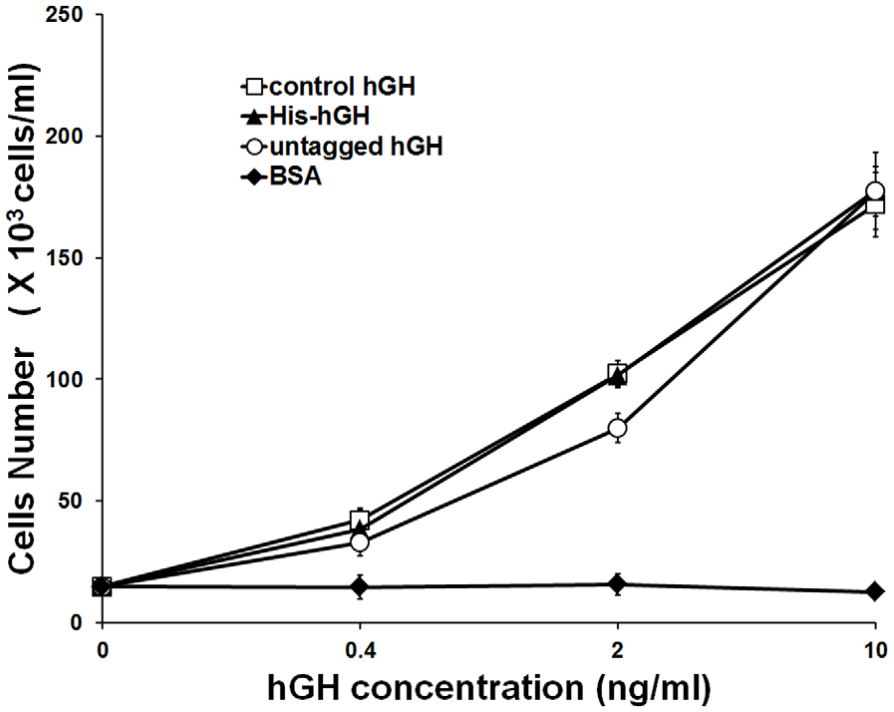
NB2-11 cell proliferation assay of purified hGH. Nb2-11 cells were arrested by serum deprivation and then incubated for 48 h in the presence of control hGH (□), His-hGH (▴), untagged hGH (○), or BSA (◆) at the indicated concentrations. Cell numbers were determined as described in Material and Methods, and averages from three independent experiments are graphed and presented as mean ± standard error.

To determine whether the purified hGH proteins were biologically active, we performed a rat Nb2-11 lymphoma cell proliferation assay [26]. Cells in starvation media were treated with different concentrations of purified His-hGH or untagged hGH, and then their growth-promoting activity was evaluated. As shown in Figure 6, both proteins greatly stimulated the proliferation of Nb2-11 cells in a dose-dependent manner in concentrations ranging from 0.4 to 10 ng/ml. Moreover, the stimulation levels of both proteins were comparable to that of control hGH. In contrast, no growth stimulation was observed with BSA, which was used as a negative control. Therefore, the hGH proteins purified under our established conditions retained their biological activity.

## Discussion

*E. coli* has been efficiently used for the high-yield production of many recombinant proteins. However, high-level expression often leads to insoluble protein aggregates such as inclusion bodies, and thus requires additional steps including solubilization and refolding prior to purification. Although the formation of inclusion bodies has certain advantages such as convenient isolation and protection from proteolysis, the recovery of biological activity has often been unsatisfactory. Furthermore, the resolubilization of protein aggregates can be problematic, requires the use of high concentrations of denaturants, and the subsequent refolding process generally requires extensive optimization.

In this study, we demonstrated the efficient preparation of soluble hGH directly from the *E. coli* cytoplasmic space under optimized conditions where protein aggregation was minimized. Although we found that a lowering of the induction temperature significantly decreased the formation of inclusion bodies, considerable amounts of recombinant hGH were still found in the insoluble pellet fraction. Unlike inclusion bodies produced by induction at high temperature, we were able to solubilize most of the pelleted hGH under our optimized protein extraction conditions. This observation suggests that they are reversible protein aggregates or clusters, rather than inclusion bodies, probably resulting from non-covalent protein interactions. Numerous detergents are known to disrupt such protein interactions. While anionic and cationic detergents are likely to disrupt protein structure, non-ionic detergents including Triton X-100 generally do not disrupt protein structure. We showed that the proper use of non-ionic detergents significantly increased the solubility of hGH during protein extraction. The solubilizing effects of such detergents can be attributed to an association with hydrophobic parts of hGH, disrupting non-specific intermolecular hydrophobic interactions. Since detergent-dependent protein solubility varies with the nature of the protein and experimental conditions, an appropriate detergent should be determined empirically.

While salt solutions (e.g., 150 mM NaCl) are commonly used for protein extraction, under our conditions the solubility of hGH was somewhat decreased by the addition of NaCl or KCl. Our observation may be explained by the fact that addition of salt leads to increased hydrophobicity on the protein surface, which results in protein aggregation through hydrophobic interactions, a process referred to as ‘salting out’ [27].

It should be noted that the soluble fraction of hGH can be destabilized and aggregated during purification. This observation often occurred with buffer exchange, which is necessary for subsequent purification steps. As glycerol is an efficient stabilizing agent for many proteins [28], we included 10% glycerol in buffers during purification and found no detectable protein aggregates.

It is known that hGH contains two intramolecular disulfide bonds, one between Cys-53 and Cys-165 and the other between Cys-182 and Cys-189 [29]. Although for some proteins disulfide bonds are crucial for their folding as reducing disulfide bonds can result in inactive protein, previous studies indicate that the disulfide bonds in hGH do not appear to be necessary for biological activity [30], [31], [32], [33]. Although hGH was expressed as a reduced form in the *E. coli* cytoplasmic space, we found that the cystein residues of hGH were spontaneously oxidized and formed intramolecular disulfide bonds under our purification conditions. Therefore, we are currently exploring whether those disulfide bonds are formed among correctly paired cysteins.

It was previously reported that the auto-induction process can be used as an efficient protein expression method [34]. Using this method, high-level expression of bubaline growth hormone was achieved, while most of the protein was found in the form of inclusion bodies [35]. Therefore, we plan to determine whether the production of hGH can be further improved by the auto-induction method combined with our established procedure for high recovery of soluble protein.

In conclusion, we describe an efficient strategy that allowed us to maximize the overall recovery of soluble hGH, which was structurally intact and biologically active. We showed that the solubility of hGH expressed in bacterial cytoplasm can be significantly increased by proper optimization of protein expression, extraction and purification procedures. Previously, prior to optimization most of the overexpressed hGH was insoluble, but under our optimized conditions we obtained up to 95% of hGH in the soluble fraction. The soluble recombinant hGH was subsequently purified by a series of chromatographic steps, and various analytical techniques confirmed the high purity, molecular mass, and structural integrity of the purified protein. Moreover, the high growth-promoting activity of purified hGH was confirmed by cell proliferation assays. Therefore, the combined effects of optimized protein expression, extraction and purification conditions significantly increased the overall recovery of soluble hGH, which is structurally intact and biologically active.

We believe that the procedures established in this work can be used by the biopharmaceutical industry to efficiently purify recombinant hGH from *E. coli* and are also worth exploring with other recombinant proteins in bacterial systems.

## Supporting Information

Figure S1 **Solubility comparison of recombinant hGH expressed at different IPTG concentrations.** The levels of soluble hGH induced at various IPTG concentrations were quantified and graphed by a densitometry assay using ImageQuant™ TL 5.2 analysis software.
(TIF)

Table S1 **Purification of His-hGH from** ***E. coli*** **.**
(DOCX)

Table S2 **Purification of untagged hGH from** ***E. coli*** **.**
(DOCX)

## References

[1] Isaksson OG, Eden S, Jansson JO (1985) Mode of action of pituitary growth hormone on target cells. Annu Rev Physiol 47: 483–499. 3888078 10.1146/annurev.ph.47.030185.002411

[2] Garcia-Barros M, Costoya JA, Rios R, Arce V, Devesa J (2000) N-glycosylated variants of growth hormone in human pituitary extracts. Horm Res 53: 40–45. 10965220 10.1159/000023512

[3] Goeddel DV, Heyneker HL, Hozumi T, Arentzen R, Itakura K, et al (1979) Direct expression in Escherichia coli of a DNA sequence coding for human growth hormone. Nature 281: 544–548. 386136 10.1038/281544a0

[4] Ikehara M, Ohtsuka E, Tokunaga T, Taniyama Y, Iwai S, et al (1984) Synthesis of a gene for human growth hormone and its expression in Escherichia coli. Proc Natl Acad Sci U S A 81: 5956–5960. 6091124 10.1073/pnas.81.19.5956 PMC391837

[5] Fahnert B, Lilie H, Neubauer P (2004) Inclusion bodies: formation and utilisation. Adv Biochem Eng Biotechnol 89: 93–142. 15217157 10.1007/b93995

[6] Datar RV, Cartwright T, Rosen CG (1993) Process economics of animal cell and bacterial fermentations: a case study analysis of tissue plasminogen activator. Biotechnology (N Y) 11: 349–357. 7763437 10.1038/nbt0393-349

[7] Khan RH, Rao KB, Eshwari AN, Totey SM, Panda AK (1998) Solubilization of recombinant ovine growth hormone with retention of native-like secondary structure and its refolding from the inclusion bodies of Escherichia coli. Biotechnol Prog 14: 722–728. 9758661 10.1021/bp980071q

[8] Singh SM, Panda AK (2005) Solubilization and refolding of bacterial inclusion body proteins. J Biosci Bioeng 99: 303–310. 16233795 10.1263/jbb.99.303

[9] Sonoda H, Sugimura A (2008) Improved solubilization of recombinant human growth hormone inclusion body produced in Escherichia coli. Biosci Biotechnol Biochem 72: 2675–2680. 18838803 10.1271/bbb.80332

[10] St John RJ, Carpenter JF, Balny C, Randolph TW (2001) High pressure refolding of recombinant human growth hormone from insoluble aggregates. Structural transformations, kinetic barriers, and energetics. J Biol Chem 276: 46856–46863. 11591710 10.1074/jbc.M107671200

[11] Becker GW, Hsiung HM (1986) Expression, secretion and folding of human growth hormone in Escherichia coli. Purification and characterization. FEBS Lett 204: 145–150. 3527743 10.1016/0014-5793(86)81403-x

[12] Gray GL, Baldridge JS, McKeown KS, Heyneker HL, Chang CN (1985) Periplasmic production of correctly processed human growth hormone in Escherichia coli: natural and bacterial signal sequences are interchangeable. Gene 39: 247–254. 3912261 10.1016/0378-1119(85)90319-1

[13] Chang CN, Rey M, Bochner B, Heyneker H, Gray G (1987) High-level secretion of human growth hormone by Escherichia coli. Gene 55: 189–196. 3311882 10.1016/0378-1119(87)90279-4

[14] Soares CR, Gomide FI, Ueda EK, Bartolini P (2003) Periplasmic expression of human growth hormone via plasmid vectors containing the lambdaPL promoter: use of HPLC for product quantification. Protein Eng 16: 1131–1138. 14983096 10.1093/protein/gzg114

[15] Bradford MM (1976) A rapid and sensitive method for the quantitation of microgram quantities of protein utilizing the principle of protein-dye binding. Anal Biochem 72: 248–254. 942051 10.1016/0003-2697(76)90527-3

[16] Jeong JS, Lim HM, Kim SK, Ku HK, Oh KH, et al (2011) Quantification of human growth hormone by amino acid composition analysis using isotope dilution liquid-chromatography tandem mass spectrometry. J Chromatogr A 1218: 6596–6602. 21840008 10.1016/j.chroma.2011.07.053

[17] Greenfield NJ (2006) Using circular dichroism spectra to estimate protein secondary structure. Nat Protoc 1: 2876–2890. 17406547 10.1038/nprot.2006.202 PMC2728378

[18] Shin NK, Kim DY, Shin CS, Hong MS, Lee J, et al (1998) High-level production of human growth hormone in Escherichia coli by a simple recombinant process. J Biotechnol 62: 143–151. 9706704 10.1016/s0168-1656(98)00054-6

[19] Patra AK, Mukhopadhyay R, Mukhija R, Krishnan A, Garg LC, et al (2000) Optimization of inclusion body solubilization and renaturation of recombinant human growth hormone from Escherichia coli. Protein Expr Purif 18: 182–192. 10686149 10.1006/prep.1999.1179

[20] Mosmann T (1983) Rapid colorimetric assay for cellular growth and survival: application to proliferation and cytotoxicity assays. J Immunol Methods 65: 55–63. 6606682 10.1016/0022-1759(83)90303-4

[21] Baneyx F, Mujacic M (2004) Recombinant protein folding and misfolding in Escherichia coli. Nat Biotechnol 22: 1399–1408. 15529165 10.1038/nbt1029

[22] Francis DM, Page R (2010) Strategies to optimize protein expression in E. coli. Curr Protoc Protein Sci Chapter 5 : Unit 5 24 21–29. 10.1002/0471140864.ps0524s61 PMC7162232 20814932

[23] Makrides SC (1996) Strategies for achieving high-level expression of genes in Escherichia coli. Microbiol Rev 60: 512–538. 8840785 10.1128/mr.60.3.512-538.1996 PMC239455

[24] Sorensen HP, Mortensen KK (2005) Advanced genetic strategies for recombinant protein expression in Escherichia coli. J Biotechnol 115: 113–128. 15607230 10.1016/j.jbiotec.2004.08.004

[25] Bondos SE, Bicknell A (2003) Detection and prevention of protein aggregation before, during, and after purification. Anal Biochem 316: 223–231. 12711344 10.1016/s0003-2697(03)00059-9

[26] Tanaka T, Shiu RP, Gout PW, Beer CT, Noble RL, et al (1980) A new sensitive and specific bioassay for lactogenic hormones: measurement of prolactin and growth hormone in human serum. J Clin Endocrinol Metab 51: 1058–1063. 7419681 10.1210/jcem-51-5-1058

[27] Baldwin RL (1996) How Hofmeister ion interactions affect protein stability. Biophys J 71: 2056–2063. 8889180 10.1016/S0006-3495(96)79404-3 PMC1233672

[28] Gekko K, Timasheff SN (1981) Mechanism of protein stabilization by glycerol: preferential hydration in glycerol-water mixtures. Biochemistry 20: 4667–4676. 7295639 10.1021/bi00519a023

[29] de Vos AM, Ultsch M, Kossiakoff AA (1992) Human growth hormone and extracellular domain of its receptor: crystal structure of the complex. Science 255: 306–312. 1549776 10.1126/science.1549776

[30] Campbell RM, Kostyo JL, Scanes CG (1990) Lipolytic and antilipolytic effects of human growth hormone, its 20-kilodalton variant, a reduced and carboxymethylated derivative, and human placental lactogen on chicken adipose tissue in vitro. Proc Soc Exp Biol Med 193: 269–273. 2320598 10.3181/00379727-193-43034

[31] Necessary PC, Andersen TT, Ebner KE (1985) Activity of alkylated prolactin and human growth hormone in receptor and cell assays. Mol Cell Endocrinol 39: 247–254. 2984068 10.1016/0303-7207(85)90068-1

[32] Dixon JS, Li CH (1966) Retention of the biological potency of human pituitary growth hormone after reduction and carbamidomethylation. Science 154: 785–786. 5919448 10.1126/science.154.3750.785

[33] Bewley TA, Brovetto-Cruz J, Li CH (1969) Human pituitary growth hormone. Physicochemical investigations of the native and reduced-alkylated protein. Biochemistry 8: 4701–4708. 5365779 10.1021/bi00840a007

[34] Studier FW (2005) Protein production by auto-induction in high density shaking cultures. Protein Expr Purif 41: 207–234. 15915565 10.1016/j.pep.2005.01.016

[35] Sadaf S, Khan MA, Akhtar MW (2007) Production of bubaline somatotropin by auto-induction in Escherichia coli. Biotechnol Appl Biochem 47: 21–26. 17087660 10.1042/BA20060154

