# Supplementary figures and images for: Complete Solubilization and Purification of Recombinant Human Growth Hormone Produced in Escherichia coli
Source: PLoS One. 2013 Feb 7;8(2):e56168. doi: 10.1371/journal.pone.0056168 (PMC3567055; doi:10.1371/journal.pone.0056168)

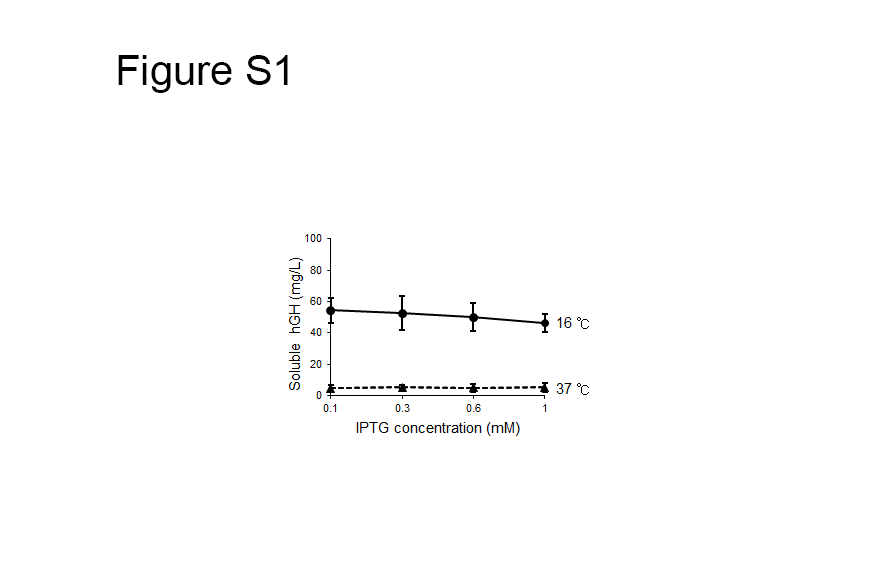

Supplement: Figure S1 — Solubility comparison of recombinant hGH expressed at different IPTG concentrations. The levels of soluble hGH induced at various IPTG concentrations were quantified and graphed by a densitometry assay using ImageQuant™ TL 5.2 analysis software. (TIF) [file pone.0056168.s001.tif]
