# Supplementary material for: Complete Solubilization and Purification of Recombinant Human Growth Hormone Produced in Escherichia coli
Source: PLoS One. 2013 Feb 7;8(2):e56168. doi: 10.1371/journal.pone.0056168 (PMC3567055; doi:10.1371/journal.pone.0056168)
Supplement: Table S1 — Purification of His-hGH from E. coli . (DOCX) [file pone.0056168.s002.docx]

**Table S1**

| **Purification step** | **Total protein**^a^  **(mg)** | **Purity of hGH**^b^  **(%)** | **hGH**^c^  **(mg)** | **Overall yield**  **(%)** |
| --- | --- | --- | --- | --- |
| Supernatant protein | 261.6 | 35.5 | 92.8 | 90 |
| Ni-NTA column | 119.6 | 70 | 84 | 81.5 |
| Mono Q column | 62.4 | 93.9 | 58.8 | 57 |
| Superdex 200 column | 40.8 | 97.9 | 40^d^ | 38.8 |

^a^Total protein was obtained from a 1 L culture and determined by the Bradford assay.

^b^The purity of hGH was determined by densitometric analysis of Coomassie blue-stained gels.

^c^The amount of hGH at each purification step was determined by the relative ratio to the total amount of protein in the particular fraction.

^d^The amount of the final product was determined by the Bradford assay.
