# Supplementary material for: Complete Solubilization and Purification of Recombinant Human Growth Hormone Produced in Escherichia coli
Source: PLoS One. 2013 Feb 7;8(2):e56168. doi: 10.1371/journal.pone.0056168 (PMC3567055; doi:10.1371/journal.pone.0056168)
Supplement: Table S2 — Purification of untagged hGH from E. coli . (DOCX) [file pone.0056168.s003.docx]

**Table S2**

| **Purification step** | **Total protein**^a^  **(mg)** | **Purity of hGH**  **(%)** | **hGH**  **(mg)** | **Overall yield**  **(%)** |
| --- | --- | --- | --- | --- |
| Supernatant | 224.8 | 37.2 | 83.6 | 86 |
| DEAE column | 126.4 | 55.6 | 70.4 | 72.4 |
| Mono Q column | 49.6 | 93.7 | 46.4 | 47.7 |
| Superdex 200 column | 34.8 | 97.2 | 34 ^b^ | 35 |

^a^Total protein was obtained from a 1 L culture and determined by the Bradford assay.

^b^The amount of the final product was determined by the Bradford assay.
